# Interferon beta-1b-induced thrombotic thrombocytopenic purpura-hemolytic uremic syndrome (TTP-HUS) in a patient treated for multiple sclerosis: A case report

**Published:** 2018-04-04

**Authors:** Masoud Etemadifar, Fatemeh Sabeti, Mehri Salari

**Affiliations:** 1 Department of Neurology, School of Medicine, Isfahan University of Medical Sciences, Isfahan, Iran; 2 Department of Neurology, School of Medicine, Shahid Beheshti University of Medical Sciences, Tehran, Iran

**Keywords:** Multiple Sclerosis, Thrombotic Thrombocytopenic Purpura, Interferon-Beta

Thrombotic thrombocytopenic purpura (TTP) and hemolytic uremic syndrome (HUS) are currently the two basic forms of thrombotic microangiopathies (TMA). Due to the extreme similarities in their clinical manifestations, they are often considered as one condition known as TTP-HUS. TTP-HUS is characterized by microangiopathic hemolytic anemia, thrombocytopenia, fever, and neurologic and renal abnormalities. Neurologic symptoms include headache, vertigo, confusion, seizures, and other symptoms. The mechanism of these symptoms is due to the obstruction of small blood vessels which causes secondary organ damage due to ischemia.^1^ Moreover, studying the literature shows that TTP-HUS has been reported in association with many inflammatory diseases and treatment with certain drugs.^2^ Multiple sclerosis (MS) is a demyelinating disease of the central nervous system which is treated with several drugs including interferon beta.^3^ Interferon beta-1a and interferon beta-1b have induced TTP-HUS in cases of patients with MS worldwide.^4^

In this study, we report a case with MS who presented with TTP-HUS. The patient went into a coma following treatment with interferon beta-1b, and then responded well to plasmapheresis and intravenous methylprednisolone.

This case report presents a 25-year-old woman with MS admitted to the emergency room on May 2016 with fever, nausea, and severely decreased consciousness. She had been diagnosed with MS in September 2014 based on some clinical manifestations including optic neuritis and paresthesia of the upper limbs and also findings of brain magnetic resonance imaging (MRI) (Figure 1) which showed MS plaques. After using 0.25 mg in 1 ml dosage of subcutaneous interferon beta-1b, her consciousness had begun to deteriorate six hours before admission to the hospital. During the next day, patient's consciousness continued to decrease, and she finally entered a comatose state, and remained in coma for the next 6 days.

**Figure 1:**
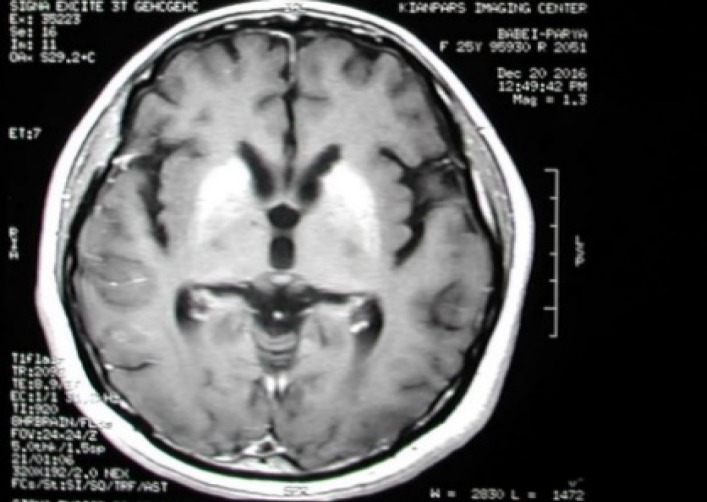
FLARE image in magnetic resonance imaging (MRI) of brain at the beginning of multiple sclerosis (MS) diagnosis

In the following 6 days after entering the coma, a series of tests were performed to indicate the cause. Her first MRI on the first day of coma indicated a diffused brain edema (Figure 2). 

**Figure 2:**
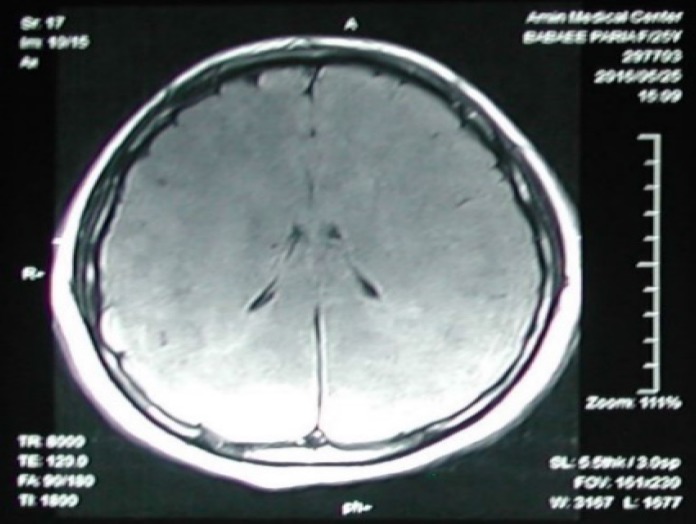
Axial FLARE image in magnetic resonance imaging (MRI) of brain shows diffused brain edema (1st day of coma)

Hematology tests indicated decreasing red blood cell (RBC) count, hemoglobin (Hb), and hematocrit (Hct). Platelet count started dropping since the first day of coma, and reached an amount under the normal range on the second day (60000 cells per cubic millimeter). By the second day of coma, lactate dehydrogenase (LDH), aspartate transaminase (AST), and alanine transaminase (ALT) levels were approximately 10, 80, and 100 times higher the normal range, respectively, which could indicate severe liver damage. DNA extracted from patient's cerebrospinal fluid (CSF) was examined through real-time polymerase chain reaction (PCR), for the presence of Herpes simplex virus 1 and 2 that came out negative. Stool exam results were completely normal. CSF analysis was normal except for LDH absolute activity which was reported 33 U/ml. 

Initially, there was an assumption of drug intoxication; however, toxicology screening test results were all negative. Since the mentioned liver enzymes were elevated and signs of kidney disorder were also observed through increased creatinine levels, and the presence of hematuria and triglyceride in urine, the patient was misdiagnosed as having hepatic encephalopathy. Following the diagnosis, the classic treatment for hepatic encephalopathy (metronidazole and lactulose) was started. After two days, considering the fact that the patient's consciousness did not improve, the diagnosis was reconsidered. The second diagnosis was TTP-HUS, considering decreased platelet counts and elevated creatinine levels. Treatment with plasmapheresis and intravenous methylprednisolone resulted in improvement of her consciousness. After 3 weeks, patient still experienced dystonic muscle spasms, especially on the right side of her body, for which treatment with botulinum toxin and physiotherapy were undertaken. Results of neuropsychiatry tests also indicated a mild cognition impairment. 

Six weeks later, another MRI was taken which indicated new MS plaques in the patient's brain. After 17 months of follow-up, muscle spasticity is completely treated; however, patient still suffers from a mild cognitive impairment. An MRI (Figure 3) was also taken which showed involvement of the basal ganglia and the diencephalon. 

**Figure 3:**
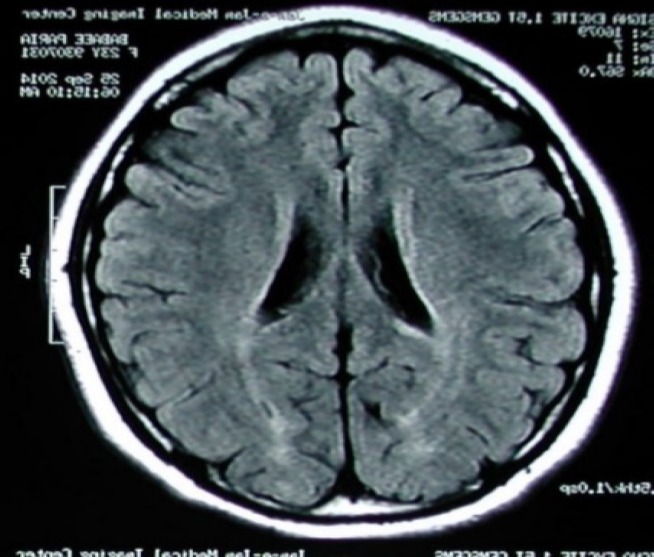
T1 weighted image in magnetic resonance imaging (MRI) of brain (six months follow-up) shows involvement of the basal ganglia and the diencephalon

There is also weakness and changes in posture in the upper right limb. Considering her last reaction to interferon beta-1b, she is now using glatiramer acetate with the dosage of 40 mg per 1 ml three times a week as a treatment for MS.

TTP and HUS are two forms of TMA which extremely overlap in their clinical manifestations. TMA cases show symptoms including a severe form of thrombocytopenia, microangiopathic hemolytic anemia, and ischemic organs, and in TTP-HUS, also renal deficiency and neurological damage.

The most common causes of acquired TTP and HUS are reported as deficiency of a disintegrin and metalloproteinase with a thrombospondin type 1 motif, member 13 (ADAMST13), the von Willebrand factor which is a cleaving protease, and infection with Shiga-toxin produced by Escherichia coli, respectively.^5^

TTP-HUS has also been observed in cases treated with certain drugs.^2^ In rare cases, treatment with intramuscular and subcutaneous interferon beta-1a, and subcutaneous interferon beta-1b in patients with MS was followed with TTP-HUS. However, the condition is much more prevalent with interferon beta-1a and only a few cases worldwide have reported TTP-HUS associated with interferon beta-1b.^3^^,^^4^

We presented a patient with MS who developed TTP-HUS during the course of her treatment with intramuscular interferon beta-1b. Patient did not show any symptoms in the first two years of using interferon beta-1b; but by the third year of treatment, she reacted negatively to the drug. After she was brought to the emergency room, she entered a comatose state, and was at first misdiagnosed with hepatic encephalopathy. In practice, TTP-HUS can easily be diagnosed with three main manifestations (triad): severely elevated LDH levels, thrombocytopenia, and schistocytosis.^2^ In our case, biochemistry tests indicated elevated serum LDH level, and nearly all hematology tests showed low platelet count; but, there was no sign of schistocytes in the peripheral blood smear. This and the elevated levels of other liver enzymes (AST and ALT) caused the doctors to miss TTP-HUS as a diagnosis. As a result, patient's improvement was delayed, and patient developed a mild cognitive impairment in addition to a permanent change in posture on the right side of her body. Had the low platelet counts been considered earlier, a full recovery of brain and motor functions after the coma could have been expected. Similarly, in a study done in Canada, a new case of a patient with MS on interferon beta-1b was reported who developed microangiopathy possibly secondary to TTP or malignant hypertension, and had not fully recovered his renal function at one-year follow up after the incident.^3^

Review of the literature indicates a certain risk of developing TTP-HUS while treatment with interferon beta, that is yet to be given the reason why. Therefore, based on our case and the previous cases reported, it is recommended that patients with MS on interferon beta be regularly monitored for any sign of abnormal platelet counts, elevated liver enzymes, and abnormal RBCs in blood smear tests for any sign of the "triad" which could indicate TTP-HUS development. Moreover, symptoms such as sudden headaches, fevers, seizures, loss of consciousness, or unexplained elevated creatinine levels should be considered critical in these patients, as they could indicate neurological or renal damage, respectively.^3^

## Notes:

**How to cite this article:** Etemadifar M, Sabeti F, Salari M. Interferon beta-1b-induced thrombotic thrombocytopenic purpura-hemolytic uremic syndrome (TTP-HUS) in a patient treated for multiple sclerosis: A case report. Iran J Neurol 2018; 17(2): 91-4.
